# Metabolomics and Lipidomics in the Study of Reproductive Performance in Livestock

**DOI:** 10.3390/ani16040588

**Published:** 2026-02-12

**Authors:** Zhengmei Sheng, Yuyang Gao, Yuqing Chong, Ying Lu, Jinpeng Shi, Huaijing Liu, Keyu Li, Weidong Deng, Jiao Wu

**Affiliations:** Yunnan Provincial Key Laboratory of Animal Nutrition and Feed, Faculty of Animal Science and Technology, Yunnan Agricultural University, Kunming 650201, China; 18008847283@163.com (Z.S.); gaoyy5210@163.com (Y.G.);

**Keywords:** metabolomics, lipidomics, reproductive performance, applied research

## Abstract

With the rapid development of omics technologies, metabolomics and lipidomics have become important tools for elucidating the molecular basis of reproductive performance in livestock. These approaches, which focus on small metabolites and lipid species, provide valuable insights into dynamic interactions between genes, proteins, and the environment, offering a high level of sensitivity. This review summarizes key methodological advances in metabolomics and lipidomics and their applications to gametogenesis, oocyte maturation, embryo development, and pregnancy maintenance. It highlights how metabolic and lipid pathways, particularly those involving energy metabolism, redox regulation, lipid remodeling, and cell–cell signaling, affect reproductive cell quality. This review further integrates biomarkers identified from semen, follicular fluid, oocytes, embryos, and uterine tissues, highlighting their potential roles in fertility assessment. Together, these insights enhance our understanding of metabolic regulation in reproduction and support the effective application of metabolomics and lipidomics in improving livestock fertility.

## Simple Summary

Reproductive efficiency is a critical factor for livestock productivity and sustainability. Recent evidence shows that metabolism not only provides energy for reproduction but also regulates gene expression through epigenetic modifications. This metabolic–epigenetic coupling affects gamete quality, embryo development, implantation, and pregnancy maintenance, but its coordination across reproductive stages is not fully understood. In this review, we summarize knowledge on metabolic–epigenetic interactions in cattle, sheep, pigs, and poultry. We focus on how metabolic pathways like glycolysis, lipid metabolism, and one-carbon metabolism interact with DNA methylation, histone modifications, and non-coding RNAs to regulate reproduction. Understanding these interactions opens new opportunities to improve fertility through nutrition, biomarker selection, and reproductive technologies, providing a framework to enhance livestock reproductive efficiency.

## 1. Introduction

Reproductive efficiency is a key determinant of genetic progress and productivity in livestock production systems. The reproductive performance of both males and females influences not only conception rates but also embryonic development, offspring viability, and the long-term improvement of herds and flocks [1]. In males, semen quality, reflected by sperm motility, morphology, membrane integrity, mitochondrial function, and genomic stability, serves as a critical indicator of fertilizing capacity [2]. High-quality sperm not only enhances fertilization rates but also ensures healthy embryonic development, thereby improving overall reproductive efficiency [3]. Likewise, in females, ovarian follicular development, oocyte maturation, cytoskeletal integrity, and mitochondrial activity largely determine the success of fertilization and early embryo development [4], making these indicators essential for evaluating reproductive outcomes.

Metabolomics and lipidomics, respectively, provide qualitative and quantitative insights into small-molecule metabolites and lipids, revealing their systemic dynamics, and have been widely applied in livestock research [5,6,7]. In livestock reproduction, metabolomics reveals energy metabolism, amino acid turnover, and redox balance critical for oocyte maturation and sperm function [8], whereas lipidomics resolves lipid class- and species-specific remodeling associated with membrane integrity, lipid droplet dynamics, and steroid hormone biosynthesis [9]. Importantly, metabolomics reflects global metabolic fluxes and cellular energetic states, while lipidomics provides structural and signaling information that is often underrepresented in conventional metabolite profiling. Integrating these approaches therefore links metabolic supply with lipid-mediated structural and signaling processes, enabling a more comprehensive characterization of reproductive metabolic states and facilitating the identification of fertility-related biomarkers and pathways relevant to assisted reproductive technologies and breeding efficiency [10]. This integrated framework provides important insights into metabolic determinants of gamete quality, embryo development, and reproductive success in livestock.

## 2. Metabolomics and Lipidomics Approaches in Reproduction Biology

Metabolomics and lipidomics, by capturing metabolic and lipid changes across reproductive cells, fluids, and tissues, offer direct insights into the biochemical states associated with fertility, gamete quality, and embryo development. Section 2 summarizes the key concepts, analytical approaches, and major platforms applied in metabolomics- and lipidomics-based reproductive research.

### 2.1. Principles of Metabolomics and Lipidomics

Metabolomics and lipidomics have become essential components of systems biology for characterizing the small molecules and lipid species that reflect the downstream outcomes of gene, protein, and environment interactions [11,12]. Metabolomics integrates high-throughput analytical and multivariate statistical approaches to systematically characterize dynamic changes in endogenous metabolites, providing insights into metabolic regulation and microenvironmental interactions [13,14]. Metabolomics can sensitively detect small-molecule metabolites in organisms, cells, tissues, and biological fluids, primarily focusing on small molecules with a molecular weight of less than 1000 Da, including key metabolic intermediates such as amino acids, organic acids, carbohydrates, and lipids [15,16]. Lipidomics employs high-throughput technologies to characterize lipid classes, structures, functions, and metabolic networks, thereby elucidating physiological and pathological lipid metabolism across cells, organs, and organisms [17,18]. The integration of lipidomics provides a complementary perspective to metabolomics, especially important when studying lipid metabolic functions [19].

The structural complexity and diversity of lipids limit the ability of conventional metabolomics to comprehensively characterize the functions, regulatory mechanisms, and dynamic interactions of individual lipid molecules. In this context, lipidomics enables precise identification and quantification of lipid molecules through highly specific analysis, thereby overcoming the limitations of traditional metabolomics in the field of lipid research [20]. Broader lipidomic coverage enables systematic analysis of lipid metabolic pathways across classes and molecular species, providing deeper insight into interactions within lipid metabolic networks [21].

These analytical approaches are increasingly valuable in reproductive biology, as metabolic and lipid remodeling underpin critical processes such as spermatogenesis, oocyte maturation, follicular development, embryo competence, and uterine receptivity [22,23].

### 2.2. Major Platforms for Profiling Reproductive Metabolites

**Figure 1:**
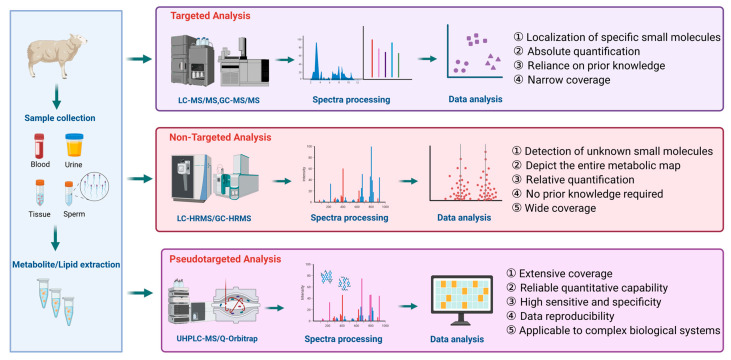
Overview of targeted, untargeted, and pseudotargeted analytical strategies in metabolomics and lipidomics. The figure highlights key differences in coverage, sensitivity, and quantitative capability, providing methodological guidance for metabolomic and lipidomic studies in livestock reproduction. In the figure, arrows indicate analytical workflow and methodological progression. Different colors represent distinct analytical strategies.

Metabolomics and lipidomics investigations generally employ untargeted, targeted, or pseudotargeted analytical strategies. Untargeted metabolomics is a hypothesis-free, global analytical strategy that enables broad and unbiased detection and identification of metabolites in biological samples without prior selection of target compounds, and is widely used to discover novel metabolic alterations and potential biomarkers [24]. Targeted metabolomics is a hypothesis-driven, validation-oriented approach that focuses on a predefined set of metabolites closely related to specific biological questions, allowing for highly accurate qualitative and quantitative analyses to verify underlying metabolic mechanisms [25]. Pseudotargeted metabolomics integrates the advantages of both untargeted and targeted metabolomics by establishing a metabolite database based on untargeted analysis and subsequently performing targeted detection of a large number of known metabolites in a single run, enabling relative or absolute quantification [26]. Together, these strategies differ in analytical workflow, metabolite coverage, sensitivity, and interpretability, as summarized in Figure 1, providing complementary tools for exploratory discovery and quantitative validation in reproductive metabolomics research [27].

Various separation and detection methods are employed in metabolomics and lipidomics experiments [28]. The most commonly used technical platforms include gas chromatography-mass spectrometry (GC-MS), liquid chromatography-mass spectrometry (LC-MS), capillary electrophoresis-mass spectrometry (CE-MS), nuclear magnetic resonance spectroscopy (NMR), high-performance liquid chromatography-tandem mass spectrometry (HPLC-MS/MS), and ultra-high-performance liquid chromatography-tandem mass spectrometry (UHPLC-MS/MS), among others [29,30]. The advantages and limitations of each analytical technique are shown in Table 1. Different technologies are not in competition but rather complement each other by providing a comprehensive global view of metabolites and lipids at a specific moment in time. Due to the diverse physicochemical properties of small molecules, a single analytical technique is often insufficient to fully cover the measurement needs of small molecules. Therefore, it is recommended to combine two or more techniques in the analysis [15].

### 2.3. Statistical and Multivariate Analysis in Metabolomics and Lipidomics

Data processing requires reliable statistical methods to effectively extract the maximum useful information. The current data analysis process in metabolomics and lipidomics includes two steps: univariate and multivariate analysis. Univariate analysis involves various statistical tests, including basic statistical tests such as *t*-tests, analysis of variance (ANOVA), as well as non-parametric tests like the Kruskal–Wallis test, to identify the most discriminative metabolites [46]. Commonly used multivariate data analysis platforms currently include principal component analysis (PCA), partial least squares discriminant analysis (PLS-DA), and orthogonal partial least squares discriminant analysis (OPLS-DA), among other multivariate statistical methods [47]. A summary of each analytical methods is shown in Table 2.

### 2.4. Integrated Metabolomics and Lipidomics in Reproductive Research

When metabolomics and lipidomics are applied together, they provide a more comprehensive view of the metabolic features underpinning reproductive efficiency. For example, the combination of membrane lipid profiling with central carbon metabolic signatures can clarify mechanisms regulating sperm motility and cryotolerance; integrating fatty acid oxidation markers with oocyte metabolic traits can improve predictions of developmental competence; and linking lipid-derived mediators with uterine metabolic transitions can enhance understanding of maternal receptivity. Thus, combined metabolomics and lipidomics analysis offers a powerful framework for elucidating fertility-associated pathways and for developing biomarkers and intervention strategies to improve reproductive performance in livestock.

## 3. Metabolic Basis of Gamete Quality and Developmental Competence in Livestock

The formation and quality of gametes in livestock are shaped by energy metabolism, mitochondrial function, lipid balance, and key molecular signals. While male and female gametes differ biologically, shared processes such as energy supply, redox balance, lipid handling, and meiotic control are central to reproductive success. Section 3 reviews recent studies showing how metabolism affects sperm function, oocyte quality, and early embryo development.

### 3.1. Metabolic Regulation of Spermatogenesis and Sperm Functional Competence

Spermatogenesis in livestock involves coordinated metabolic transitions that support germ cell proliferation, meiosis, and fertilization capacity. Immature spermatogonia primarily rely on lactate supplied by Sertoli cells for ATP production, while developing spermatocytes and spermatids shift toward glycolysis and mitochondrial oxidative phosphorylation to meet the energy demands of chromatin remodeling, meiosis, and flagellar assembly [53,54]. Mitochondrial fatty acid β-oxidation also contributes to sustaining the high ATP turnover required for continuous flagellar motility [55,56]. These energetic processes are closely linked to sperm membrane composition, particularly polyunsaturated fatty acids, which provide fluidity and fusogenic capacity but increase susceptibility to oxidative stress [57].

At the molecular level, spermatogenesis and maturation are regulated by coordinated signaling pathways, transcription factors, and small RNAs. STRA8, induced by retinoic acid (RA), triggers the expression of meiotic markers SYCP3 and γH2AX, promoting spermatogonia differentiation and ensuring spermatogenesis [58]. Cyclin-dependent kinases (CDK7) regulate RA-mediated signaling, facilitating spermatogonial proliferation, meiotic entry, and spermatocyte differentiation [59]. Proper formation of the synaptonemal complex ensures homologous recombination, while small RNAs, such as miRNAs, modulate signaling pathways including TGF-β to fine-tune spermatogenic progression. High-throughput sequencing of key miRNAs and their target genes in chicken spermatogenesis revealed that miRNA-301a-5p regulates spermatogenesis by targeting TGFβ2 in the TGF-β signaling pathway [60]. GnRH stimulates the release of LH and FSH, with LH promoting testosterone production by Leydig cells and FSH supporting Sertoli cell proliferation, both essential for spermatogenesis [61,62]. Heat stress (HS) disrupts cattle gut microbiome balance, with A. muciniphila regulating secondary bile acid metabolism, enhancing retinol absorption, increasing testicular RA-levels, and improving spermatogenesis [63]. Melatonin reshapes the gut microbiota, regulates arachidonic acid metabolism, reduces testicular inflammation, protects the blood-testis barrier, and alleviates heat stress-induced spermatogenesis disorders in dairy goats [64].

Based on the above research findings, spermatogenesis and maturation in male livestock rely on efficient energy supply, involving pathways such as glycolysis, lipid metabolism, and oxidative phosphorylation. Abnormalities in these metabolic pathways can lead to insufficient energy supply, resulting in increased oxidative stress, cell apoptosis, and membrane structural abnormalities, which significantly affect sperm production and quality. At the same time, spermatogenesis is coordinated by the synergistic regulation of genes such as *STRA8*, *CDK7*, *SYCP3*, and various metabolic pathways, ensuring the orderly progression of each stage of sperm development in male livestock.

### 3.2. Metabolic Regulation of Follicular Development and Oocyte Maturation

The follicle, consisting of the oocyte and surrounding somatic cells like granulosa and theca cells, is the basic functional unit of the ovary and essential for female reproductive capacity [65]. Follicular development and oocyte maturation require significant energy, with glycolysis and the TCA cycle playing critical roles. Impaired enzyme activity in these pathways can disrupt oocyte maturation [66]. Lipids in oocytes serve as both energy substrates and precursors for steroid hormone synthesis, supporting follicular development, oocyte maturation, embryo development, and uterine function [67,68]. For example, β-aminoisobutyric acid (BAIBA) supplementation enhances fatty acid β-oxidation, mitochondrial activity, and lipid metabolism, improving bovine oocyte quality [69].

Omics technologies reveal key genes, metabolites, and metabolic pathways involved in follicular development and oocyte maturation. For example, the RA signaling pathway, with its downstream target *STRA8*, initiates meiosis, marking the transition from mitosis to meiosis in germ cells [70]. *PDE3A* regulates oocyte meiosis, with its inhibition leading to elevated cAMP levels, causing oocyte arrest at the germinal vesicle (GV) stage [71]. The RNA-binding protein CPEB3, in collaboration with miR-15/16, regulates cyclin E1 mRNA translation, ensuring proper cell cycle progression during oocyte maturation. Its absence results in follicular development arrest and ovarian dysfunction [72]. *CDK1* and *Cyclin B1* form the MPF complex, regulating oocyte meiosis resumption. *RSPO2* promotes granulosa cell proliferation and *CDK1* expression via the Wnt pathway, supporting follicular development and oocyte maturation [73,74]. Kisspeptin, encoded by *KISS1*, binds to GPR54 on GnRH neurons, stimulating GnRH release and inducing estrus and ovulation in livestock [75]. RNA-seq analysis of follicles from goats of different birth types revealed that miRNAs like miR-10b and miR-100 regulate ovulation rate and litter size by targeting genes like BMP15 and GDF9, affecting pathways such as PI3K-Akt, TGF-β, and steroidogenesis [76]. RNA-seq analysis of the ovaries in Tibetan sheep revealed that lymphatic system marker genes such as *LYVE1*, *ADAMTS-1*, and LH/FSH signaling-related genes like *EGR1*, *FKBP5*, *DUSP1*, and *FOS* were all downregulated, leading to follicular development arrest [77]. In chickens, silencing LPL reduces lipid droplet content and inhibits expression of lipid metabolism and steroidogenesis genes, impairing follicular development [78].

These findings highlight that follicular development and oocyte maturation are regulated by a sophisticated molecular network involving genes, transcription factors, lipids, metabolites, proteins, and multiple signaling pathways.

### 3.3. Metabolic Pathways Shaping Early Embryo Development and Implantation

Early embryo development and pregnancy maintenance, which involve complex metabolic regulation, are essential for reproductive efficiency in livestock, and balanced dietary lipid and fatty acid intake helps reduce early embryonic loss. The metabolism of phospholipids, sphingolipids, and cholesterol promotes embryonic development and implantation through molecules such as endocannabinoids, prostaglandins, and steroid hormones [79]. In sheep, integrated metabolomic and transcriptomic analyses have identified key metabolites, including glutamine and phosphatidylcholine, together with genes such as DNMTs, PCNA, and GLUTs that are associated with oocyte maturation and embryo development. These findings provide a molecular basis for optimizing in vitro embryo production and improving reproductive efficiency [80]. Embryo-maternal communication occurs via extracellular vesicles (EVs), with miRNAs in EVs regulating gene expression at the embryo-maternal interface, influencing embryo growth and implantation [81]. Metabolomics analysis in high-producing sows found that levels of S-adenosylmethionine (SAM) and homocysteine (Hcy), linked to methionine metabolism, regulate birth weight and placental angiogenesis [82]. In ewes, lipidomic and metabolomic profiling of the uterine cavity and embryos on day 14 of pregnancy revealed elevated ceramides, diglycerides, fatty acids, and phospholipids, as well as increased amino acids and carbohydrates in the uterine cavities. These metabolic changes support embryo growth and elongation during early pregnancy [83]. Proteomic analysis in sheep placentas showed that placental development is regulated by dynamic protein expression networks, involving pathways like PI3K-Akt, MAPK, and estrogen signaling. Key regulatory factors (*SRC*, *MAP3K1*, *KRAS*, *TJP1*) exhibit stage-specific expression, ensuring successful pregnancy [84]. Pregnancy in livestock is regulated by hormones (progesterone, interferon-tau, placental lactogen, estrogen) and metabolic pathways, including one-carbon metabolism, PPP, serine synthesis, and polyamine metabolism, which support uterine development, placental function, immune balance, and nutrient supply [85]. In pigs, chemerin, an adipokine, regulates uterine angiogenesis by upregulating *VEGF-A/B*, *PlGF*, and *VEGFR3* while downregulating *VEGF-C/D*, *ANG-1*, and AMPK phosphorylation, particularly in early pregnancy [86]. RT-PCR analysis of buffalo uterus during early pregnancy showed that LPA receptors *LPAR1* and *LPAR6*, particularly *LPAR6*, are upregulated in the endometrium, promoting early pregnancy establishment and maintenance [87].

**Figure 2:**
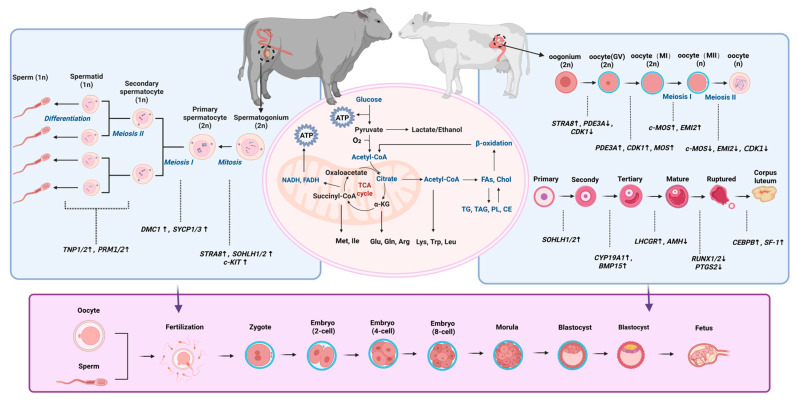
Integrated regulation of gametogenesis and early embryonic development by energy metabolism and coordinated multi-gene signaling pathways. The schematic illustrates how metabolic pathways interact with signaling networks to support gamete development and early embryonic competence. In the figure, upward arrow indicates upregulation and down arrow indicates downregulation. Different colors represent distinct functional modules or regulatory sections.

The genesis and maturation of germ cells in both male and female livestock represent highly energy-consuming and dynamic processes that depend on efficient metabolic energy supply and coordinated regulation by multiple genes and signaling pathways (Figure 2). These processes lay the foundation for early embryo development and the establishment and maintenance of pregnancy, which are further governed by lipid metabolism, signaling molecules, nutritional substrates, extracellular vesicles, and stage-specific gene and protein networks. Together, these factors orchestrate embryo–maternal communication, placental function, and uterine receptivity, ultimately determining reproductive efficiency in livestock.

## 4. Metabolic–Epigenetic Coupling in Livestock Reproduction

The coupling of metabolic processes and epigenetic regulation is a critical mechanism determining gametogenesis, embryo programming, pregnancy maintenance, and offspring health in livestock [88,89]. Key metabolites such as SAM, acetyl-CoA, ATP, NAD^+^, and α-ketoglutarate (α-KG) act as cofactors or substrates for DNA and histone modification enzymes, regulating chromatin accessibility and DNA methylation patterns, thus influencing oocyte maturation, sperm quality, embryo genome activation, and pregnancy establishment [90]. Through this metabolic–epigenetic interface, environmental and nutritional cues are translated into heritable molecular signals that shape oocyte maturation, sperm quality, embryo genome activation, and pregnancy establishment across species.

SAM, generated through one-carbon metabolism, serves as the universal methyl donor for DNMTs and histone methyltransferases, directly determining DNA and histone methylation intensity. Nutrients such as folate, methionine, betaine, choline, and vitamins B_6_ and B_12_ regulate intracellular SAM availability and thereby influence epigenetic programming during gametogenesis and early embryogenesis [91,92]. DNA methylation patterns were examined at different stages of embryo development, especially during the early stages involving two major epigenetic reprogramming events. These studies revealed that DNA methylation plays a crucial role in embryo viability and fetal development, closely linked to various metabolic and differentiation processes [93,94]. In sheep, in vitro embryo culture studies demonstrated that betaine contributes methyl groups via the methionine–cysteine cycle and helps regulate osmotic balance, while combined supplementation with L-carnitine enhanced oocyte developmental competence, highlighting a direct link between one-carbon metabolism, methylation capacity, and embryo quality [80]. In Japanese Black cows, maternal nutrient restriction (MNR) notably altered DNA methylation patterns and gene expression in the fetal thymus, particularly genes related to metabolism (*MIF*, *GPX1*, *GPX4*, *NDUFS8*, *NDUFB10*, *PC*, *IDUA*, *ACAD8*, *CYC1*, *GGT7*) and immunity (*PTPN11*, *PAK1*) [95]. Similarly, reduced methionine availability in bovine granulosa cells and embryos induced genome-wide DNA methylation changes affecting more than 1600 genes, including imprinted genes associated with fetal overgrowth syndrome, such as *PEG10* and *IGF2R* [96]. Furthermore, maternal methionine deficiency in ducks affected the mRNA abundance of one-carbon metabolism genes (*GLRX*, *BHMT*, *BHMT2*, *DHFR*, *MAT2*, *RBBP4*, *HNF4A*) and energy metabolism genes (*ELOVL6*, *GPAM*, *NDUFA4*, *PGM1*) in newly hatched offspring, thus impacting their development and health [97]. During porcine follicular development, genes with differential m6A methylation in granulosa cells were significantly enriched, potentially linked to steroidogenesis and folliculogenesis [98]. Moreover, sperm in livestock carry various RNAs, including mRNA fragments and regulatory RNAs such as siRNA, miRNA, piRNA, and lncRNA, which provide epigenetic signals that influence embryo development and regulate gene expression in the offspring [99]. α-KG is an essential cofactor for TET family–mediated DNA demethylation as well as for JmjC domain–containing histone demethylases [100]. Evidence indicates that insufficient α-KG availability or abnormal accumulation of succinate suppresses the enzymatic activities of TET and JmjC demethylases, resulting in incomplete DNA demethylation and aberrant retention of H3K27me3, thereby impairing zygotic genome activation (ZGA) and early embryonic developmental kinetics [101]. Beyond DNA demethylation, acetyl-CoA and NAD^+^ regulate reproductive processes by modulating histone acetylation and deacetylation. Acetyl-CoA serves as the direct acetyl donor for histone acetyltransferases (HATs), and its intracellular availability reflects cellular energy metabolic status. Through regulation of activating histone marks such as H3K9ac and H3K27ac, acetyl-CoA participates in oocyte maturation, meiotic progression, and zygotic genome activation (ZGA) [102]. In bovine in vitro fertilization (IVF) and embryo culture systems, insufficient glucose or pyruvate supplementation in the culture medium limits acetyl-CoA synthesis, leading to a marked reduction in H3K9ac and H3K27ac levels. This epigenetic alteration is associated with delayed ZGA, reduced blastocyst formation rates, and compromised embryo quality [103].

**Figure 3:**
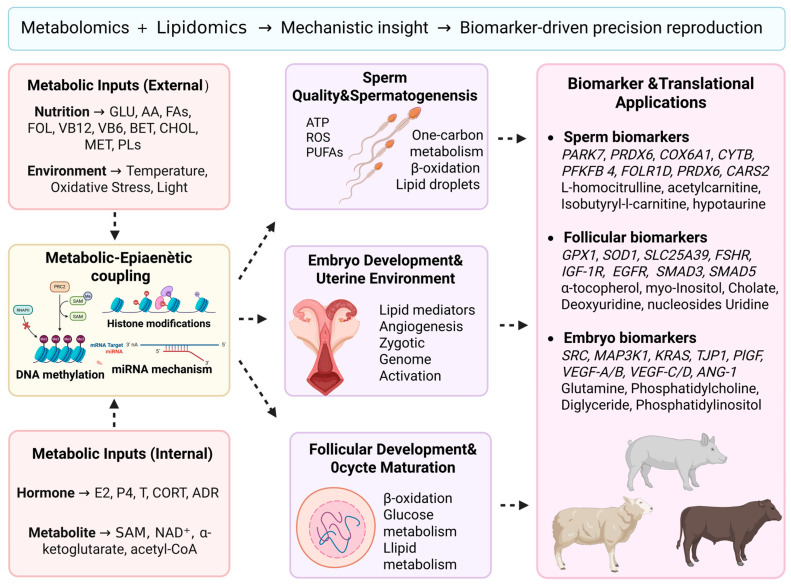
Linking metabolomics and lipidomics to metabolic–epigenetic regulation of reproduction and biomarker-driven precision breeding. The figure summarizes how metabolomic and lipidomic insights inform metabolic–epigenetic regulation and support the identification of fertility-related biomarkers for precision breeding. In the figure, different colors represent distinct functional modules, and arrows indicate regulatory or functional interactions between components.

These mechanistic insights provide a conceptual basis for linking metabolomic and lipidomic profiles to fertility-related biomarkers and precision reproductive strategies (Figure 3).

## 5. Evaluating Biomarkers for Gamete Quality and Fertility in Livestock

In livestock, the quality of gametes plays a key role in fertility. Omics approaches can detect biomarkers in semen, follicular fluid, oocytes, and oviductal fluid providing insights into changes in energy metabolism, motility, viability, and oxidative stress for evaluating gamete health.

### 5.1. Biomarkers for Assessing Male Livestock Germ Cell Quality

Multi-omics research has identified key biomarkers reflecting the oxidative balance, metabolic activity, and developmental potential of male germ cells in livestock. Proteomic and metabolomic analysis of frozen-thawed sperm from HMS and LMS Gaoqing bulls showed that high levels of proteins like PARK7 and PRDX6, along with metabolites such as L-homocitrulline, acetylcarnitine, and isobutyryl-L-carnitine, help regulate reactive oxygen species (ROS) during freezing, preserving sperm vitality and preventing oxidative stress [104]. A GWAS analysis of Duroc boars identified SNPs and genes (*SLC10A6*, *MYRF*, *GGA1*, and *UTRN*) linked to sperm cryotolerance, helping to identify boars with high sperm freezing tolerance and improve semen cryopreservation efficiency [105]. LC-MS analysis of Duroc boar seminal plasma identified metabolites such as D-proline, L-citrulline, and leucine during storage at 17 °C, which were enriched in amino acid synthesis and metabolism–related pathways, supporting an association between amino acid metabolism and sperm liquid preservation ability [106]. GC-MS analysis of Holstein bull sperm identified metabolites involved in alanine, glutamate, and β-alanine metabolism, providing key indicators for fertility potential [107]. Lipidomic analysis of Cobb broiler semen plasma, sperm cells, and whole sperm identified lipids like phosphatidylserine and sphingolipids that correlate with sperm motility, offering insights into poultry sperm quality [108].

Environmental factors significantly impact sperm quality and fertility. Untargeted metabolomics revealed that HS increased cortisol levels and decreased testosterone, impairing spermatogenesis. Metabolites like L-tryptophan and kaempferol were downregulated, further affecting sperm fertilization potential [109]. In Bos indicus, sperm exhibited more efficient energy metabolism, higher membrane integrity, and lower ROS levels, enhancing fertility in tropical environments [110]. In Tibetan sheep, high-altitude adaptation was linked to regulation of cAMP pathway genes (*ADCY*, *PRKACA*) and metabolites like adenosine and prostaglandin I2, helping maintain sperm vitality [111]. Table 3 shows representative biomarkers for evaluating the reproductive cell quality and fertility of male livestock.

The above studies have identified a series of key biomarkers closely related to sperm energy metabolism, motility, antioxidant capacity, and developmental potential. These biomarkers cover multiple dimensions, including oxidative stress, genetic factors, functional proteins, and metabolic lipids. Not only do these biomarkers accurately reflect the physiological status of sperm, but they also provide potential molecular selection targets for breeding practices.

### 5.2. Biomarkers for Assessing Female Livestock Germ Cell Quality

The quality of female reproductive cells, especially oocytes, is crucial for fertilization, early embryo development, and long-term reproductive success. Using omics technologies to identify biomarkers of oocyte quality at the molecular and functional levels enhances our understanding of reproductive mechanisms in livestock. Antioxidant defense is a key mechanism for ensuring oocyte quality. In sows, glycine increased the expression of antioxidant genes like *GPX4*, *CAT*, *SOD1*, *SOD2*, and *SLC25A39* while boosting glutathione (GSH) levels, reducing lipid peroxidation, and supporting oocyte maturation [117]. Similarly, in bovine follicles, progesterone levels were associated with changes in cumulus and granulosa cell gene expression and miRNA profiles in follicular fluid sEVs, with upregulation of ADAMTS-1 and AGO2 potentially influencing oocyte maturation [118]. RT-PCR analysis of cumulus cells and oocytes in prepubertal gilts and sows revealed changes in *BBOX1*, *CPT2*, *G6PD*, and *ALDOA* expression, identifying these genes as potential biomarkers for oocyte quality [119]. In Holstein cows, the expression of *FSHR*, *IGF-1R*, *AMH*, *EGFR*, *Bax*, and *Caspase-3* in cumulus cells was linked to oocyte developmental competence [120]. Shotgun proteomics analysis of pig follicular fluid identified SERPINE, PLAU, and PLAUR as stage-specific proteins involved in follicle size regulation and maturation, serving as biomarkers for oocyte quality [121]. In Angus cattle, higher pre-ovulation estradiol levels were positively associated with ATP content and mitochondrial DNA copy number in oocytes, suggesting that a favorable follicular environment may promote oocyte development [122].

Collectively, antioxidant molecules, FF metabolites, miRNA gene regulatory pairs, and lipid metabolic enzymes provide complementary layers of information for evaluating oocyte developmental competence across livestock species. To consolidate these findings, representative biomarkers associated with oocyte competence, follicular metabolism, and female reproductive performance across livestock species are summarized in Table 4.

### 5.3. Applications and Translational Value

Evaluating biomarkers of sperm and oocytes in livestock reproduction management can assist breeders in more accurately selecting breeding animals with high reproductive potential. This not only improves fertilization rates and embryo developmental success but also optimizes the preservation techniques for frozen semen and oocytes. In the identification of sheep sperm proteins, it was found that the expression level of the sperm structural protein ProAKAP is significantly positively correlated with sperm vitality and cryotolerance. Notably, ProAKAP can effectively reflect sperm functional integrity, even after cryopreservation, making it a valuable biomarker for optimizing sperm preservation protocols [127]. A widely targeted metabolomic analysis of Hu sheep testicular tissue revealed that well-developed testes exhibit significantly elevated expression of genes associated with multiple energy metabolism pathways. These include glycolysis (GLTU8 and LDH), TCA cycle (PDHA2, CS, and IDH3G), gluconeogenesis (PCK1), the pentose phosphate pathway (G6PD), and fatty acid degradation (GK, ACSL1, FABP3, CPT1, and CPT2). This suggests that enhanced energy metabolism contributes to testicular development [128]. Dietary or follicular fluid-specific fatty acids, such as α-linolenic acid and cis-9, trans-11 conjugated linoleic acid, influence granulosa cell function in Holstein cows. They downregulate steroidogenesis-related genes, including STAR and CYP19A1, and reduce the production of hormones such as estradiol and progesterone, potentially impairing follicular development and oocyte quality [129]. Moreover, SNPs in lipid metabolism genes such as *CHKA*, *GNAI1*, *HMOX2*, *CAT*, *MYOF*, and *RBP4*, which are associated with pregnancy extension, have been confirmed to be significantly correlated with traits such as daughter pregnancy rate and first-service conception rate in Holstein cows. These genetic markers can be used for genomic selection to improve reproductive efficiency in cattle [130]. Adding *GDF8* to the culture medium of in vitro fertilized pig embryos can improve mature oocyte quality by regulating p38 mitogen-activated protein kinase phosphorylation and intracellular GSH and reactive oxygen species levels [131].

The translational application of biomarkers can not only enhance reproductive success in livestock but also provide new approaches and technical support for improving breeding programs and reducing the risk of reproductive failure.

## 6. Integrated Synthesis and Future Perspectives

The collective evidence synthesized in this review demonstrates that metabolism provides the unifying biochemical foundation linking gamete quality, embryonic competence, and the establishment of pregnancy in livestock. These cross-stage metabolic dependencies can be distilled into several conserved regulatory themes.

### 6.1. Conserved Metabolic Pathways Underpinning Reproductive Cell Competency

Across gametogenesis, fertilization, and early embryogenesis, a set of conserved metabolic pathways repeatedly emerges as the biochemical foundation of reproductive success in livestock. Glycolysis and mitochondrial oxidative phosphorylation ensure ATP availability for spermatogonial proliferation, oocyte maturation, and embryo cleavage, while β-oxidation supports sperm motility and fuels trophoblast elongation [132]. Lipid remodeling, including the regulation of PUFA-containing phospholipids, sphingolipids, and cholesterol esters, maintains membrane stability in gametes and generates bioactive mediators such as prostaglandins, endocannabinoids, and lysophosphatidic acid that guide implantation and uterine remodeling [79]. Amino acid metabolic pathways, particularly glutamine metabolism and one-carbon cycle, provide essential substrates for biosynthesis and regulate SAM-dependent methylation reactions. Transcripts for most enzymes involved in these metabolic pathways are expressed in ovarian follicles, oocytes, and pre-implantation embryos of cattle, sheep, and pigs, thereby linking nutrient status with the epigenetic regulation of development during reproduction [96]. These fundamental metabolic axes recur across male germ cells, oocytes, embryos, and the pregnant uterus, highlighting metabolism as the primary integrator of reproductive cell function.

### 6.2. Metabolism–Epigenetics Axis as a Regulatory Core

Accumulating evidence indicates that metabolic pathways not only provide cells with energy and raw materials but also influence gene expression through the regulatory effects of their products. This, in turn, regulates key reproductive processes at the epigenetic level, such as oocyte maturation, embryo development, and placental function [133]. Through epigenetic mechanisms like methylation and histone modifications, metabolic intermediates such as SAM, Hcy, lipids, and amino acids shape adaptive gene expression patterns during different developmental stages [134]. These mechanisms not only help the organism adapt to environmental changes but also play vital roles in embryonic growth and development, immune regulation, and energy metabolism.

Although some studies have revealed the fundamental role of the metabolism–epigenetics axis in livestock and poultry reproduction, the specific molecular mechanisms remain unclear. Future research should further explore the interplay between different metabolic pathways and epigenetic modifications, elucidating how environmental factors, nutritional interventions, or genetic mutations regulate reproductive processes through metabolic signals. Furthermore, with advancements in single-cell genomics and metabolomics technologies, researchers will be able to more accurately depict the dynamic changes in the metabolism–epigenetics axis. By precisely regulating metabolic pathways or epigenetic markers, new strategies for improving reproductive efficiency in livestock may emerge, thereby enhancing agricultural productivity and promoting sustainable resource use. At the same time, research based on the metabolism–epigenetics axis could provide theoretical support for addressing climate change, improving stress resistance, and optimizing animal health.

### 6.3. Opportunities for Metabolomics-Driven Interventions

Although substantial progress has been made in defining the metabolic determinants of gamete quality, embryonic development, and pregnancy establishment, several research gaps remain. First, most current studies examine isolated stages (sperm, oocytes, embryos, or placenta), yet reproductive success depends on the coordinated metabolic continuity across these stages. Longitudinal, cross-stage multi-omics analyses are needed to establish causal links between gamete metabolism, embryo competence, and placental function. Second, dynamic metabolic profiling remains limited. Techniques such as single-cell metabolomics, stable isotope tracing, and spatial lipidomics could provide higher-resolution insights into how metabolic fluxes change in real time during early development. Third, the impact of environmental challenges, including heat stress, high-altitude hypoxia, nutritional fluctuations, on reproductive metabolism is increasingly relevant for livestock systems. Studies in Tibetan sheep already highlight metabolic adaptations involving adenylate cyclase, prostaglandin pathways, and ATP-regulated signaling [111], but broader cross-species investigations are needed to support resilience breeding. Finally, integrating metabolic markers into reproductive management, through optimized embryo culture media, targeted nutritional supplementation, or early pregnancy diagnostics, represents a promising direction for improving fertility in livestock.

## 7. Conclusions

Importantly, metabolomic and lipidomic studies offer strong translational potential for livestock reproduction by identifying metabolic signatures linked to gamete quality, embryo viability, and uterine receptivity. These metabolic insights provide a foundation for improving assisted reproductive technologies, embryo selection, and nutritional and reproductive management strategies. Moreover, targeted modulation of key metabolic pathways through diet, metabolic programming, or culture system optimization represents a practical approach to enhance reproductive efficiency and offspring health. Future research should focus on integrating metabolomics and lipidomics with other omics approaches to achieve a systems-level understanding of reproductive regulation and to support the development of precision reproductive and management strategies for sustainable livestock production.

## Figures and Tables

**Table 1:** Comparison of commonly used analytical platforms in metabolomics and lipidomics.

Technology	Advantages	Limitations	References
GC-MS	High resolution; high selectivity; high sensitivity; broad target coverage	Suitable mainly for volatile compounds; derivatization required for non-volatile metabolites	[29,31]
LC-MS	High throughput; high flexibility; high sensitivity; broad metabolite coverage	Ion suppression and matrix effects; limited structural information; difficulty in absolute quantification	[9,29,31,32,33]
CE-MS	High resolution; high sensitivity; low sample and reagent consumption	Lower robustness; limited sensitivity for some analytes; charge-dependent separation constraints	[29,31,34]
NMR	Inherent quantitativeness; high repeatability; non-destructive analysis	Low sensitivity; limited resolution; high operational cost	[19,29,31]
HPLC-MS/MS	High sensitivity; high repeatability; strong specificity; multi-analyte capability	Limited by analyte stability and ionization efficiency; moderate throughput	[35,36,37,38,39,40]
UHPLC-MS/MS	Ultra-high separation efficiency; high sensitivity; reduced matrix interference; reliable method validation	High instrument cost; complex sample pretreatment and data processing	[41,42,43,44,45]

**Table 2:** Commonly used multivariate statistical methods in metabolomics and lipidomics.

Method	Features	Limitations	References
PCA	Unsupervised method for dimensionality reduction and data visualization	Assumes linear relationships; cannot directly handle categorical variables	[48,49,50]
PLS-DA	Supervised method for classification and discrimination in high-dimensional data	Risk of overfitting; relatively high computational complexity	[48,49,51]
OPLS-DA	Supervised extension of PLS-DA that separates predictive and orthogonal variation, improving interpretability	Requires high data quality; increased model complexity	[49,51,52]

**Table 3:** Metabolite-, protein-, and lipid-based biomarkers associated with sperm quality and fertility in male livestock.

Breed	Technologies	Biomarker	Function	References
Gaoqing bulls	Proteomics, Untargeted metabolomics	PARK7, PRDX6, L-homocitrulline, acetylcarnitine, Isobutyryl-l-carnitine	Prevent sperm from oxidative stress and apoptosis	[104]
Landrace boars	Non-Targeted Metabolomics, Proteomics	*COX6A1*, *CYTB*	Involved in alterations in the level of the metabolites in boar X/Y sperm	[112]
Holstein Friesian Crossbred bulls	Transcriptomics, Proteomics, Metabolomics	*PFKFB 4*, *IPMK*, *FOLR 1D*, *DNM 2*, *EEF 2*, *PRDX 6*, *CARS 2*	Regulates pathways such as Butanoate metabolism, Glycolysis and gluconeogenesis and so on	[113]
Boar	RT-qPCR, WB	AQP4, AQP6, AQP3, AQP7, AQP10	Expression levels of AQPs-mRNA could modify sperm homeostasis	[114]
Holstein bulls	Lipidomics	SFA, MUFA, PUFA, BCFA	Correlates with membrane integrity, fluidity, and stability	[115]
zebu bulls, Hariana × Holstein-Friesian	Non-targeted metabolomics	taurine, hypotaurine, phosphatidylcholine, phosphatidylethanolamine	Regulates taurine, hypotaurine and glycerophospholipid metabolism, modulates sperm quality	[116]

**Table 4:** Metabolite-, protein-, and gene-based biomarkers associated with oocyte quality and follicular development in female livestock.

Breed	Technologies	Biomarker	Function	References
Osmanabadi goats	Untargeted metabolomics	α-tocopherol, GPX1	Improves oocyte maturation by reducing reactive oxygen species	[123]
post-mortem sows	Shotgun proteomics	SERPINE, PLAU, PLAUR	Regulates oocyte maturation in follicle sizes	[121]
Angus cattle	RNA-seq metabolomics	GnRH2, progesterone, estradiol, LH	Induced ovulation, correlates with oocyte developmental competence	[122]
Lohmann Layer	Transcriptomics	*SMAD3*, *SMAD5*, *ID1*, *ID2*, *ID3*	Correlates with follicular development	[124]
STH sheep	RT-qPCR	*BMPR1B*, *BMP15*, *GDF9*	Promote follicular growth and maturation	[125]
Jersey cows	metabolomics	myo-Inositol, Cholate, nucleosides Uridine, Deoxyuridine, N-acetyl-D-glucosamine 6-phosphate	Correlates with oocyte maturation and ovulation	[126]

## Data Availability

No new data were created or analyzed in this study. Data sharing is not applicable to this article.
